# Polymeric Micelles Enhance Mucosal Contact Time and Deposition of Fluocinolone Acetonide

**DOI:** 10.3390/polym14112247

**Published:** 2022-05-31

**Authors:** Sucharat Limsitthichaikoon, Siriwat Soontaranon, Nuntachai Hanpramukkun, Kanjana Thumanu, Aroonsri Priprem

**Affiliations:** 1 Department of Pharmaceutical Technology, College of Pharmacy, Rangsit University, Pathum Thani 12000, Thailand; nuntachai.h@rsu.ac.th; 2 Synchrotron Light Research Institute, Nakhon Ratchasima 30000, Thailand; siriwat@slri.or.th (S.S.); kanjanat@slri.or.th (K.T.); 3 Faculty of Pharmacy, Mahasarakham University, Maha Sarakham 44150, Thailand; apriprem@gmail.com

**Keywords:** drug–mucin contact time, focal plane array (FPA)-based Fourier transform infrared (FTIR) imaging, hydrophobic drug, mouth rinse, polymeric micelles

## Abstract

This study used polymeric micelles to improve quality by increasing drug solubility, extending mucosal drug retention time, enhancing mucoadhesiveness, and promoting drug permeation and deposition. Fluocinolone acetonide (FA) was loaded into polymeric micelles (FPM), which were composed of poloxamer 407 (P407), sodium polyacrylate (SPA), and polyethylene glycol 400, and their physicochemical properties were examined. Small-angle X-ray scattering (SAXS) revealed a hexagonal micellar structure at all temperatures, and the concentrations of P407 and SPA were shown to significantly affect the solubility, mucoadhesion, release, and permeation of FPMs. The proportion of P407 to PEG at a ratio of 7.5:15 with or without 0.1% *w*/*v* of SPA provided suitable FPM formulations. Moreover, the characteristics of FPMs revealed crystalline states inside the micelles, which was consistent with the morphology and nano-hexagonal structure. The results of ex vivo experiments using focal plane array (FPA)-based Fourier transform infrared (FTIR) imaging showed that the FPM with SPA penetrated quickly through the epithelium, lamina propria, and submucosa, and remained in all layers from 5–30 min following administration. In contrast, the FPM without SPA penetrated and passed through all layers. The FPM with extended mucoadhesion, improved drug–mucosal retention time, and increased FA permeation and deposition were successfully developed, and could be a promising innovation for increasing the efficiency of mouth rinses, as well as other topical pharmaceutical and dental applications.

## 1. Introduction

Common mucocutaneous diseases, such as oral lichen planus, may arise separately or simultaneously in the oral cavity [1]. Although the cause of many oral lesions is unclear, there is evidence to support an association with immune function [1]. The standard treatment for oral lichen planus is corticosteroids, including fluocinolone [2] as an oral paste, gel, or mouth rinse [3]. Pastes or gels are more commonly used due to their mucoadhesive properties [2]; however, it can be difficult to ensure adhesion of the dosage to some oral lichen planus lesions. Topical mouth rinses are less mucoadhesive, but are better able to disperse, flow, and access hidden areas of the lesions. Moreover, topical mouthwash provides a level of therapeutic efficacy similar to other commercial oral lichen planus treatments [4]. Solutions often struggle to permeate pseudomembranes, achieve drug–mucosal contact time, and rapidly clear the formulation. As such, strategies that enhance the permeation and deposition of active ingredients into the mucosal tissues are most desirable. 

Polymeric micelles have been identified as one of the most promising systems for the delivery of active ingredients, particularly for Biopharmaceutics Classification System (BCS) class II and IV drugs, and can enhance drug deposition into tissues [5,6]. Nano-polymeric micelles have also been shown to be kinetically stable carriers, combining amphiphilic block copolymers as a core–shell that can hold hydrophobic drugs within a hydrophilic medium [7]. Not only do micelle-forming copolymers improve the solubility of active compounds with poor water solubility, but they also increase the stability of drugs in polymeric micelles [8]. Poloxamer is one polymer used to prepare these micelles, and it is applied in drug delivery formulations [9]. Poloxamer can also be mixed with polyethylene glycol (PEG) to help enhance drug absorption [10]. Moreover, the modification of poloxamer by adding an acrylate group enhances the mucoadhesive and increases micelle–mucus interaction [7], a finding that has increased the interest in polymeric micellar innovation. To create an ideal nanocarrier, the ratio of polymers must be suitably optimized.

From recent evidence, fluocinolone acetonide (FA) is the standard drug used to treat oral lichen planus; however, oral mouth rinses are significantly less effective, as they have reduced drug–mucosal contact time due to decreased mucoadhesion, and the deposition of the drug to basal cells as steroid target receptors. This study therefore developed solutions of polymeric micelles to improve mucoadhesion, extend drug–mucosal retention time, and increase FA deposition. FA polymeric micelles were fabricated using factorial design, and their physicochemical properties (including mucoadhesion, in vitro release, in vitro permeation, ex vivo drug accumulation, and drug stability) were examined in order to acquire a better understanding of polymeric micelles’ organization and their impact on the penetration of lipophilic drugs into the mucosa.

## 2. Materials and Methods

### 2.1. Materials

Fluocinolone acetonide (FA) (Farmabios, Pavia, Italy) was provided as a gift from Siam Chemi-Pharm (1997) Co., Ltd, Bangkok, Thailand. Poloxamer 407 (P407), sodium polyacrylate (SPA), and mucin type II from porcine stomachs (lot No. SLCC7713) were purchased from Sigma-Aldrich^®^, St. Louis, MO, USA. Polyethylene glycol 400 (PEG) was purchased from Aketong Chemipun, Bangkok, Thailand.

### 2.2. Preparation of Polymeric Micelles Loaded with FA

#### 2.2.1. Formulation Development

Polymeric micelles loaded with FA (FPMs) were created by incorporating 0.1% *w*/*v* of FA into polymeric micelles composed of P407 (%*w*/*v*) and PEG (%*w*/*v*), with or without SPA (%*w*/*v*). The concentrations of these compounds are shown in Table 1. P407 was dissolved in a phosphate buffer saline (PBS) solution of pH 7.4 and left to absorb completely overnight at 4 °C, before being mixed with FA in PEG using a magnetic stirrer at 28–32 °C. A solution of SPA in PBS with a pH of 7.4 was eventually added, if applicable. A solution of PBS (pH 7.4) was then added for final volume adjustment.

#### 2.2.2. Blank Polymeric Micelles 

The blank polymeric micelle (BPM) solution was prepared according to the same method as the FPMs (without the addition of FA) and was labeled as BPMs.

#### 2.2.3. Dried Powder of FPMs and BPMs

The FPM and BPM solutions were lyophilized at −80 °C and 1 bar using a freeze dryer (Modulyo benchtop freeze dryer, Thermo Electron Corporation, MA, USA) for 2 days to obtain the dried powder.

### 2.3. Physicochemical Characteristics 

#### 2.3.1. Particle Size, Polydispersity Index, and Zeta Potential 

A dynamic light scattering (DLS) NanoPlus^®^ (NanoPlus-3, Serial No. 409314, Boulder, CO, USA) instrument was used to measure the particle size, polydispersity index (PDI), and zeta potential of the formulations, using the nanoparticle size and zeta potential analyzer modes. Each FPM formula was pre-treated by dispersal in distilled water prior to measurement (n = 6). 

#### 2.3.2. Study of Polymeric Micelle Solutions Using Small Angle X-ray Scattering

Small angle x-ray scattering (SAXS) measurements were carried out at the Synchrotron Light Research Institute, Thailand. The synchrotron light generated from a multipole wiggler source was monochromatized using a double multilayer monochromator to provide the X-ray energy of 9 keV. Throughout the SAXS measurements, the sample was contained in a temperature-controlled sample holder with a Kapton window. The sample-to-detector distance was 4563 mm and was calibrated using a styrene–ethylene–butadiene–styrene (SEBS) standard block polymer. The SAXS patterns were recorded using a Rayonix SX165 CCD detector. A 1D SAXS profile was obtained by normalizing the scattering pattern with beam intensity, X-ray transmission, and background subtraction using the software provided by in-house software at SLRI (SAXSIT) at the beamline. The polymeric micellar structure of FPM samples fixed at 4, 25, and 37 °C was observed.

#### 2.3.3. Viscosity and Mucoadhesion

Viscosity (η, cps) of the FPMs (n = 6) was measured using a viscometer (Brookfield Model DV-II+ viscometer; MA, USA) at room temperature (25 ± 5 °C) and 100 rpm. 

Mucoadhesiveness was assessed by observing the interactions of FPMs and mucin type II and measuring the bond strength between the polymeric micelle solution and the glycoproteins in the mucus [11,12], according to the results of our previous study [13]. Each FPM (5 mL) was mixed with mucin type II (5 mL) in test tubes, and the tube was inverted 5 times without shaking. Each mucin-formulation mixture was then measured for viscosity at 100 rpm, and at 0, 15, 30, and 60 minutes (min) without re-shaking. The percentage of mucoadhesiveness was analyzed using the Equation (1):

(1) $$\%\;\mathrm{Mucoadhesiveness}=\frac{\mathrm{average}\;\eta \;\mathrm{of}\;\mathrm{mixture}\;-\;\mathrm{average}\;\eta \;\mathrm{of}\;\mathrm{formulation}}{\mathrm{average}\;\eta \;\mathrm{of}\;\mathrm{formulation}}\times 100\%$$

#### 2.3.4. Fourier Transform Infrared Spectroscopy

The dried FPM samples were analyzed using Fourier transform infrared spectroscopy (FTIR) to determine functional groups and changes in their molecular fingerprints. The FPMs that had been lyophilized or combined with a mucin sample were mixed with potassium bromide under 10 tons of hydraulic pressure, and an FTIR spectrometer (PerkinElmer Inc., Spectrum One program, Waltham, MA, USA) was used to record the IR spectra in the region from 4000–400 cm^−1^.

#### 2.3.5. Thermal Analysis Using Differential Scanning Calorimetry, Thermal Gravimetric Analysis, and Powder X-ray Diffraction Analysis

Each powder sample was placed in a pierced aluminum pan, and heated at a scanning rate of 10 °C per min from 25 °C to 300 °C using a nitrogen flush, at a rate of 20 mL/min. A blank aluminum pan was used as a reference. Thermographs were then obtained and were analyzed using a differential scanning calorimeter (DSC) 8000 (PerkinElmer, MA, USA) to determine melting points and transition temperatures (exothermic or endothermic). A simultaneous thermal analyzer (STA) 6000 (PerkinElmer, USA) was used to determine physical changes in the materials by monitoring weight loss on heating. 

A thermal gravimetric analyzer (TGA, Simultaneous thermal analyzer [STA] 6000 [PerkinElmer, USA]) was employed with a cooling machine in a nitrogen bath (20 mL per min) to analyze 5 mg samples in a pre-weighed aluminum pan. The pan was covered with a lid and heated at a constant rate of 20 °C per min from 25 °C to 400 °C. 

The powder X-ray diffraction analysis (PXRD) pattern was performed by using the MiniFlex II diffractometer (Rikaku, Tokyo, Japan) and recorded at 30 kV and 15 mA in the 2θ range. The temperature was increased from 5 °C to 45 °C at a rate of 5 °C per min using Cu Kα radiation (λ = 0.154 nm). 

#### 2.3.6. Microscopic Morphology

Samples were pretreated by dropping onto a copper grid with a carbon film coating (200 mesh copper) and were then air dried. The samples were viewed using high resolution field emission transmission electron microscope (FE-TEM, TALOS F200, Thermo Fisher Scientific, Waltham, MA, USA). 

#### 2.3.7. Prelimination Stability Testing

All FPM formulae were stored at 3 different temperatures (4 °C, 25 °C, and 45 °C) to determine their stability. At days 7 and 30 of storage, sample pH and FA percentages were determined and compared with those of freshly prepared samples. 

### 2.4. In Vitro Release and Permeation

In vitro release experiments were carried out using the dialysis tubing experiment. Each FPM solution contained in a 5 mL syringe and weighing approximately 5 g, was loaded into a 35 × 50 mm dialysis membrane tube (dialysis membrane standard RC Tubing MWCO 3.5K Da, Spectra/Por^®^, Waltham, MA, USA), avoiding the gas bubble and tided with dialysis tubing clamps with magnetic stirrers. Each dialysis bag was placed into a closed container, contained 250 mL of artificial saliva (composed of 0.17% sodium bicarbonate, 0.05% sodium dihydrogen orthophosphate, and 0.02% calcium chloride in water at pH 7.4 [13]), and was stirred at 600 rpm at 37 ± 2 °C.

In vitro permeation experiments were also performed using Franz diffusion cells (Crown Glass Co., Somerville, NJ, USA.). The non-keratinized mucous membrane of the porcine esophagus was used as the barrier membrane [14,15]. The esophagus, purchased from a local slaughterhouse (Rangsit, Pratum Thani, Thailand), was cut open longitudinally, and the membranes soaked in normal saline solution at 60 °C for 45 s. The epithelial mucous layer was isolated by peeling it off from the underlying connective tissue. The mucosal membrane had a thickness of approximately 350 ± 18 μm and was washed thoroughly with PBS pH 7.4. It was then used immediately.

Each FPM solution contained in a 5 mL syringe and weighing approximately 5 g and was loaded into the donor chamber, and the receptor chamber was filled with 1% bovine serum albumin solution (Sigma-Aldrich, USA) in phosphate buffer at pH 7.4 to maintain a sink condition [16]. This was stirred at 600 rpm at 37 ± 2 °C. 

A measure of 0.5 mL of receptor medium was collected at 5, 15, 30, 60, 120, 240, 360, and 480 min, and replaced with an equal amount of fresh receptor medium. The percentage of FA was analyzed at a wavelength of 254 nm using a UV-VIS spectrophotometer (Shimadzu^®^, Model UV-1800, Tokyo, Japan). The cumulative amount of total FA that permeated from the polymeric micelle solution was calculated as a percentage of the total FA content of the relevant donor.

### 2.5. Ex Vivo Drug Accumulation

The barrier membranes of the porcine esophagus used in the in vitro permeation studies that were exposed to FPM7 and FPM8 for 5, 15, and 30 min were fixed at −80 °C with an optimal cutting temperature (OCT) reagent (OCT Tissue-Tek^®^, Torrance, CA, USA). The esophageal membranes were then cross-sectioned using a microtome to a thickness of approximately 8–10 mm and width of 1 mm. The cross-sections were placed on a barium fluoride (BaF_2_) crystal slide, vacuum dried, and observed under a focal plane array (FPA)-based fourier transform infrared (FTIR) imaging (FPA FTIR; FTIR microscope with a 20× Ge Lens [HYPERION 3000, Bruker Optics, Billerica, MA, USA]), and with an FPA imaging detector [mapping measurement]) using transmission mode with a 36× lens, integrated within the wavenumber range of 4000–800 cm^−1^ for 3D images to characterize intensities of the IR spectra. This detected 64 scans with a spectral resolution of 4 cm^−1^ and 4 × 4 binning. 

### 2.6. Statistical Analysis

Categorical variable data were evaluated as percentages (n = 6). Continuous variable data were described as averages and standard deviations (SD), and the normality was tested. Student *t*-test and analysis of variance (ANOVA) were performed to examine differences between or among experimental groups using SPSS 13 software (SPSS Inc., Chicago, IL, USA). A *p*-value of < 0.05 was taken as statistical significance, and a one-way ANOVA was used to compare the average values.

## 3. Results and Discussion

### 3.1. Effects of Independent Variables on Characterization of Polymeric Micelles

The FPM formulations containing 0.1% *w*/*v* of FA and varied quantities of P407 (5–10% *w*/*v*) and PEG (5–15% *w*/*v*), with or without 0.1% *w*/*v* SPA, were compared in 18 formulations, as shown in Table 1. The concentrations of P407, PEG, and SPA in the polymeric micelles affected the appearance, particle size, and zeta potential of the formulation. The FPM1–6 solutions appeared white and turbid, while the FPM7–14 solutions were clear. The FPM15–18 solutions showed precipitation. Solutions composed of high concentrations of P407 and PEG were turbid and had a large particle size of formulation (shown in Table 1). The appearance of FPM solutions was also associated with particle size and the PDI, which was also related to the concentrations of P407, PEG, and SPA. A high concentration of P407 consisted of large particle sizes, as shown by the micrometer range, and appeared as a white suspension (turbid) instead of clear. A low concentration of P407 created small composite polymeric micelles. However, the addition of SPA to FPM formulations produced a higher particle size than the absence of SPA. 

The zeta potential of the formulations containing SPA was found to be negatively charged, while formulations without SPA were positively charged. The proportion of P407 to PEG at a ratio of 7.5:15 provided the highest zeta potential for both negative and positive charges. 

Following 7 days of storage, FPM1, 2, 7, 8, 13, and 14 remained homogeneous mixtures, in which the ratio of P407:PEG could affect the stability of the micellar solution of the hydrophobic drug in hydrophilic solution [7,17]. These concentrations of P407 act as stabilizers, which could facilitate the solubility of poorly water-soluble molecules and form self–micelle aggregates with other co-solvents of polymers, thus increasing the FA solubility [18,19].

**Figure 1:**
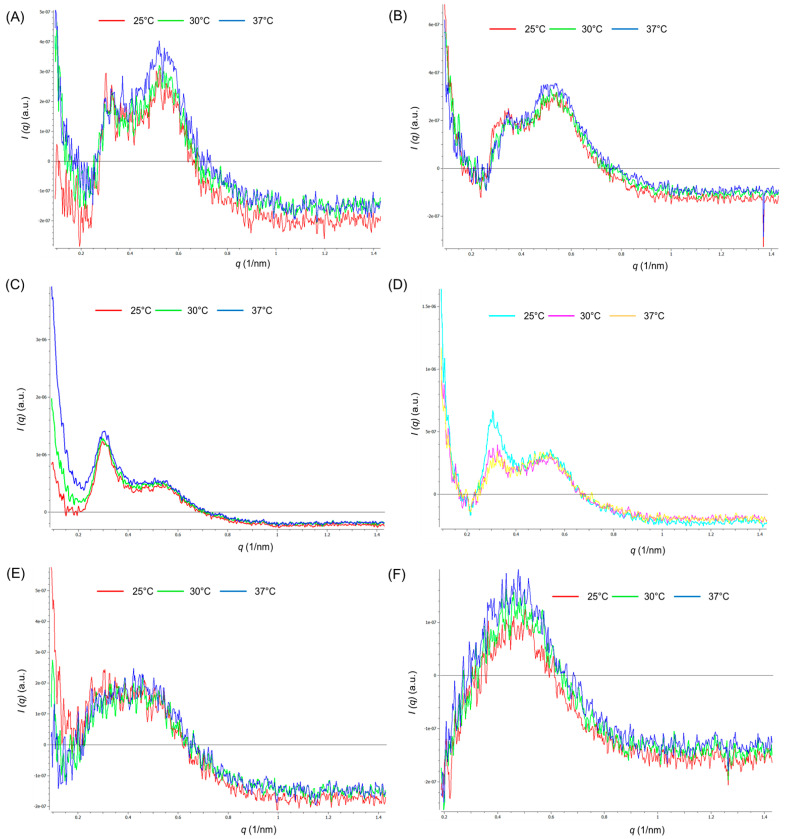
SAX structure of fluocinolone acetonide polymeric micelles (FPMs) 1 (**A**), 2 (**B**), 7 (**C**), 8 (**D**), 13 (**E**), and 14 (**F**) for all temperatures as 25 °C, 30 °C and 37 °C.

The micellar structures of FPM1, 2, 7, 8, 13, and 14 were observed by the SAXS profile (Figure 1). The hexagonal structure could be determined from the peak position ratio 1:√3:2:√7 for the first, second, third, and fourth peaks in the SAXS profile, respectively [20,21]. FPM1 and FPM2 showed poorly ordered hexagonal structures at all temperatures (25 °C, 30 °C, and 37 °C). FPM7 maintained a similar hexagonal micellar structure at all temperatures. FPM8 also had hexagonal micellar structure at all temperatures; however, from the higher amplitude of the first peak at 25 °C, it is likely that micelles had a higher-order hexagonal structure at this temperature compared to micelles at 30 °C and 37 °C. The arrangement of P407 might affect the polymeric micelles structure at temperatures above 25 °C [22,23]. The first peak at q = 0.3 found in FPM13 and FPM14 could not be observed, making it difficult to confirm whether this system has a hexagonal structure at all temperatures. The hexagonal structure of micelles was dependent on the concentration of P407, in relation to the ratio of PEO–PPO–PEO interaction [24] and the complex conformation of P407 and PEG in the mixtures [25]. Determining the micellar structures at different temperatures was carried out to (1) identify polymeric micellar structures according to various concentrations of P407 and the presence of SPA [7]; (2) examine the effects of diverse temperature on micellar structure and stability [26]; and (3) determine the drug carrier delivery type of FPMs [27].

### 3.2. Effects of Independent Variables on Responses in Design Formulation

**Figure 2:**
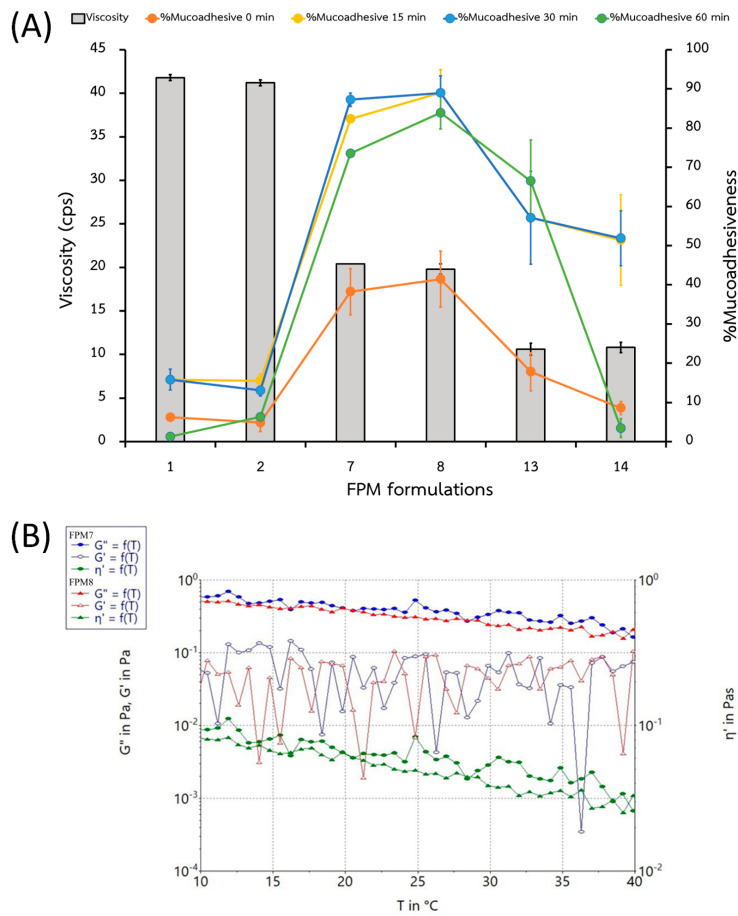
Comparison (**A**) of the formulation viscosity (orange columns) and % mucoadhesiveness at 0 (grey line), 15 (yellow line), 30 (blue line) and 60 min (green line) and (**B**) rheology of FPM7 and FPM8 between 10 °C to 40 °C.

The maximum concentrations of P407 and PEG provided the greatest viscosity for FPM1 and FPM2 (Table 2 and Figure 2A); in contrast, the presence of SPA did not significantly affect their viscosity or the percentage of mucoadhesiveness. The impact of P407 on FPM viscosity might be explained by the nature of the self-molecule micelle, which acts as a reservoir in a polymeric matrix [28]. However, increasing the P407 ratio was not found to increase the percentage of mucoadhesiveness (Figure 2A), as FPM7 and FPM8 showed the greatest formula–mucin bonds, and the duration of formula–mucin interaction was highest at 0 and 15 min (Table 2). The highest mucoadhesion (%) for all FPMs was observed at 15 min and decreased following 30 and 60 min of bonding. As for the use of mouth rinse, low mucoadhesiveness may not provide the retention time required for treatment or relief of symptoms. Thus, 15 min of formula–mucin contact time with the binding provided a suitable in vitro method for evaluating this factor.

The FPM1–6 composed of high concentration of P407 (10%) observed thermoreversible sol-gel transition when exposed to high temperatures (greater than 45 °C) due to the properties and the concentration of P407 [18,23,28]. Phase separation at 25 °C was observed in FPM3–6 and FPM15–18. P407 is widely used in pharmaceutical technologies to increase hydrophobic solubilization and drug stabilization. This includes for in situ gel formulation, which is used to fabricate each dosage form depending on the PEO and PPO units of poloxamer and other polymers [29]. Thus, using these composite polymers and concentrations, polymeric micelles and aggregation were found.

The varying temperatures may affect sol-gel transition but did not correspond with the viscosity and rheology of the polymeric micelles in FPM7 and FPM8 (Figure 2B). Hydrophilic or amphiphilic block copolymers (such as P407 and SPA) undergo self-assembly into polymeric micelles, with insoluble drugs [7] inside a hydrophobic core, surrounded by hydrate shell layers [30]. Sol-gel transition did not occur due to the character of the polymeric micelles and concentration of polymers in FPM7 and FPM8. This was confirmed by the SAXS profiles, which revealed their hexagonal structure, the result of the resistance of micellar structures to rearrangement at high temperatures (25 °C, 30 °C, and 37 °C) [21].

### 3.3. Percentage of FA Release and Permeation in Vitro Studies

**Figure 3:**
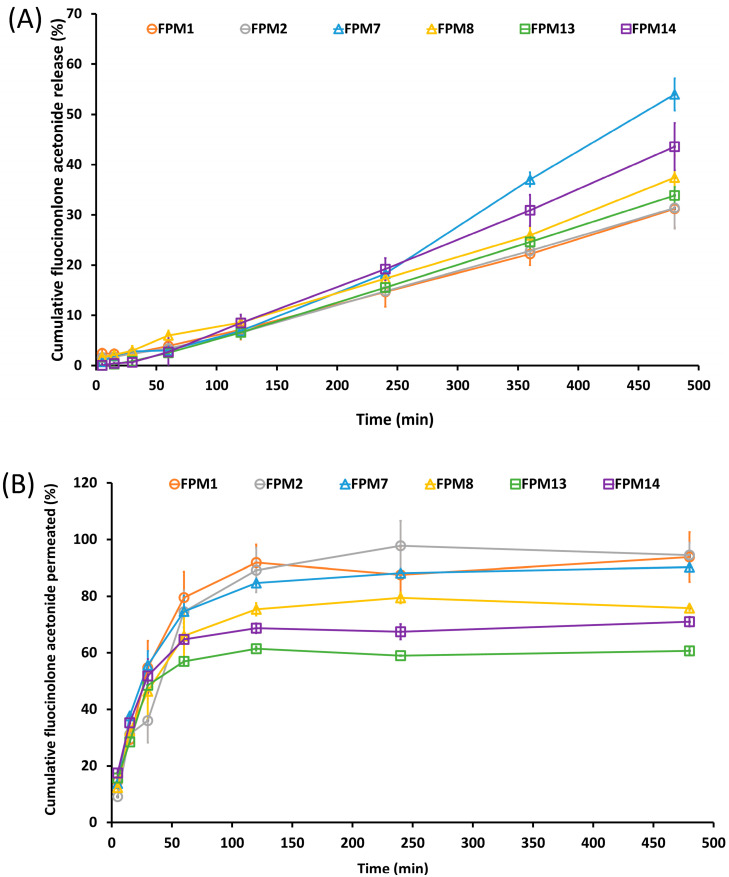
Percentage of fluocinolone acetonide (FA) release (**A**) and percentage of fluocinolone acetonide (FA) permeation (**B**) of all FPMs formulation.

The cumulative release of FA from FPMs was investigated in vitro over 8 h, as shown in Figure 3A. This amount of time was used to assess the phase action of mouth rinse formulations for treatment of oral lichen planus, which is required 3 times daily [1]. An FA burst effect is therefore essential, as well as mucoadhesiveness and drug penetration. The percentage of the cumulative amount of FA release and permeation at the initial 5 min has been investigated as shown in Table 2. Even though the FA release profile of FPM7 and FPM8 showed slow release in a saliva medium with about 1.70 ± 0.001 and 0.76 ± 0.003% of drug release, respectively, the permeation rate, which was 17.76 ± 0.001 and 12.37 ± 0.03%, was shown to be higher than the other formulations. These results might be caused by the effect of P407 and the formation of micelles and enhanced FA delivery into the mucosa [19,31].

FA release and permeation were examined using kinetic models such as zero-order, first-order, Higuchi, and Korsmeyer-Peppas models (Table 3 and Table 4). Zero-order kinetic models were plotted as the cumulative amount of drug released over time (as shown in Equation (2)). First-order kinetic models were plotted as the logarithmic cumulative amount of drug released over time (as shown in Equation (3)). Higuchi models were plotted as the cumulative amount of drug released over the square root of time (as shown in Equation (4)). Korsmeyer-Peppas model describe diffusion mechanisms of the proportion of drug diffusion at time (as shown in Equation (5)):

(2) $$Q\;={\;k}_{0}t\;$$

(3) $$\ln\;Q\;={\;\mathrm{lnQ}}_{0}-k_{1}t$$

(4) $$Q\;={\;k}_{H}t^{1/2}$$

D_t_ / D_∞_ = K_KP_ t^n^ (5)

Q represents the amount of drug released at a time, while Q_0_ is the initial drug concentration. k_0_ represents the rate constant corresponding to the zero-order model, and k_1_ describes the rate constant corresponding to the first-order model. k_h_ is the rate constant corresponding to the Higuchi order model. t is the time in hours, and t^1/2^ is the square root of the time. D_t_ / D_∞_ represented the proportion of drug diffusion at time t, K_KP_ is the kinetic constant, and n is the diffusion exponent. 

The drug release profile over 480 min was analyzed using linear regression, as shown in Table 3. All FPM formulations were fitted to the zero-order, first-order, Higuchi, and Korsmeyer-Peppas models. FA release followed Korsmeyer-Peppas kinetics, as the r^2^ approached 1, indicating that the FA release of FPMs was provided polymeric matrix drug delivery [32]. When considering the diffusion exponent (n-value) of the Korsmeyer-Peppas’s equation, the n-values were n > 1 [33]. Therefore, the drug is released in a super case II manner. The release of FA from the formulation was from both diffusion and erosion of the polymeric micelle’s matrix [28]. The ideal duration of drug release for treatment of oral lichen planus depends on the area of exposure, mucoadhesion, particle size, drug carrier, and drug release within 8 h. All FPM formulations showed a Korsmeyer-Peppas release pattern, and FPM7 had the highest release rate (Figure 3 and Table 3).

The 8-h in vitro permeation is shown in Figure 3B. Although FPM1 provided the highest percentage of FA release, FPM7 had the greatest percentage of FA permeation. Moreover, the mucoadhesiveness of mucin to formulations of FPM7 was directly related to the FA permeation, due to the drug–mucin interaction. For use of mouth rinse, which has minimal contact time between the formula and the mucosal surface, the mucoadhesiveness of FPM7 promoted FA penetration into the mucosa [3]. The linear regression of the drug permeability profile was analyzed within 60 min (Table 4), and all FPM formulations were fitted to zero-order, first-order, Higuchi, and Korsmeyer-Peppas models. [32]. The Korsmeyer-Peppas model was the best fit for FA permeation as the r^2^ approached 1, indicating that FPMs provided a polymeric matrix drug diffusion [32,33]. When considering the diffusion exponent (n-value) of the Korsmeyer-Peppas’s equation, n-values were in the range of 0.45 < n < 0.89 [33]. Therefore, the drug is permeated in a non-Fickian diffusion manner. The permeation of FA from the FPM was from both diffusion and erosion of the polymeric matrix [34] and demonstrated drug permeation by a diffusion mechanism.

The release profile of the formulations in saliva showed slow release but high permeation through the epithelial layer within the first hour. The high mucoadhesiveness promoted contact with the epithelium, which increased the amount of FA that permeated through the epithelial layer. FPM1, FPM2, and FPM7 showed similar permeation rates, with FPM7 exhibiting high mucoadhesiveness and permeation, and slow release in saliva in the first hours, according to the hexagonal micellar structure formation from the polymeric surfactant. The interaction between the side chain of the polymeric surfactant and glycoproteins in the mucus layer was highly mucoadhesive [7] and involved diffusion mechanisms from the hexagonal micellar structure [20,35]. FA follows BCS class II, which acts to facilitate low solubility and high permeation. Thus, the contact time of mucoadhesion between FPM7 and mucin allowed FA to permeate the epithelium.

### 3.4. Prelimination Stability Testing

All FPM formulations were stored in a desiccator at 3 different temperatures (4 °C, 25 °C, and 45 °C) to determine FA stability. All formulations retained the same pH after 30 days of storage. The percentage of FA contents (shown in Table 5) reveals that storage at 45 °C affected the remaining FA, while the percentage of FA remained the same when stored at 4 °C and 25 °C. The percentage of drug content of FPM7 and FPM8 was found to be the stable for 30 days at all temperatures. The hexagonal micellar structure of FPM7 and FPM8 may therefore serve as a barrier to prevent FA from contacting water in the external medium.

### 3.5. Polymeric Micellar Characteristics and Physicochemical Properties

**Figure 4:**
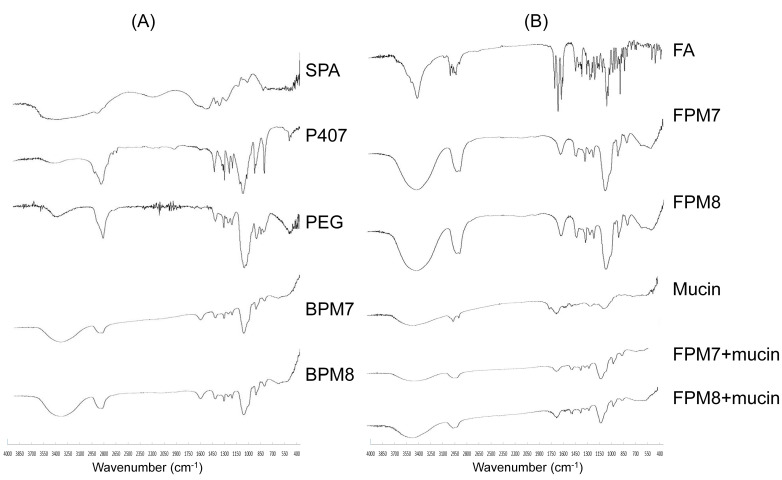
Representative FTIR spectra of (**A**) sodium polyacrylate (SPA), poloxamer 407 (P407), and polyethylene glycol 400 (PEG) compared to the blank polymeric micelles (BPM7 and BPM8) and (**B**) fluocinolone acetonide (FA) compared to FPM7 and FPM8, mucin type II and formulation with mucin.

To determine FA–polymeric micellar interactions, FTIR, XRD, DSC, and TGA were performed. FTIR spectra (Figure 4) revealed changes in drug–excipient functional groups caused by the interaction. The FTIR of SPA showed a principle peak at 1600 cm^−1^, representing the C=O group, while the FTIR of PEG had a peak at 3500 cm^−1^ for O–H, at 2800 cm^−1^ for C–CH_3_, and at 1200 cm^−1^ for C–O–C. P407 also had peaks at 3500 cm^−1^ for O–H, 2800 cm^−1^ for C–CH_3_, 1280 cm^−1^ for a C–H stretch aliphatic, 1340 cm^−1^ for a plane O–H bend, and 1100 cm^−1^ for a C–O stretch. After composing polymeric micelles, the functional groups of BPs still showed key peaks at 3500 cm^−1^ for the O–H group and 1200 cm^−1^ for the C–O–C group. FA showed sharp peaks at 3500 cm^−1^ for the O–H group, 2700–2900 cm^−1^ for C–H and C–CH_3_ cm^−1^, 1600 cm^−1^ for an aromatic ring, and 1200 cm^−1^ for C–O–C. FTIR results showed no differences in peaks between FPM7 and FPM8, for which the strong bands (such as the carbonyl peak and aromatic ring) were visible in the formulation, and this indicates that no chemical interaction occurred during the synthesis of FPMs. 

Lyophilization of formulations and mucin was performed by FTIR analysis, to observe changes in functional group interactions. Mucin showed dominant peaks at 2700–2900 cm^−1^ for C-H and C-CH_3_ cm^−1^, and at 1200 cm^−1^ for C-O-C (Figure 4). The formula–mucin mixture showed a reduction in peaks at 3500 cm^−1^ (O-H group) and 1200 cm^−1^ (C-O-C group), which suggests a covalent bond interaction between FPMs and mucin. Moreover, an OH band shift was observed, which could relate to hydrogen bonding. The formula–mucin bonding of FPMs was not very strong, and this could be due to hydrogen bonding between hydroxyl and carboxyl groups [11]. The non-ionic bonds of the formula–mucin complex could be involved, but the negative charges of the formulas and mucin provide weak bonds that may decrease over time [13]. Furthermore, external forces or stimuli (such as shaking) could create a stronger formula–mucin interaction, and this factor is likely essential in application.

**Figure 5:**
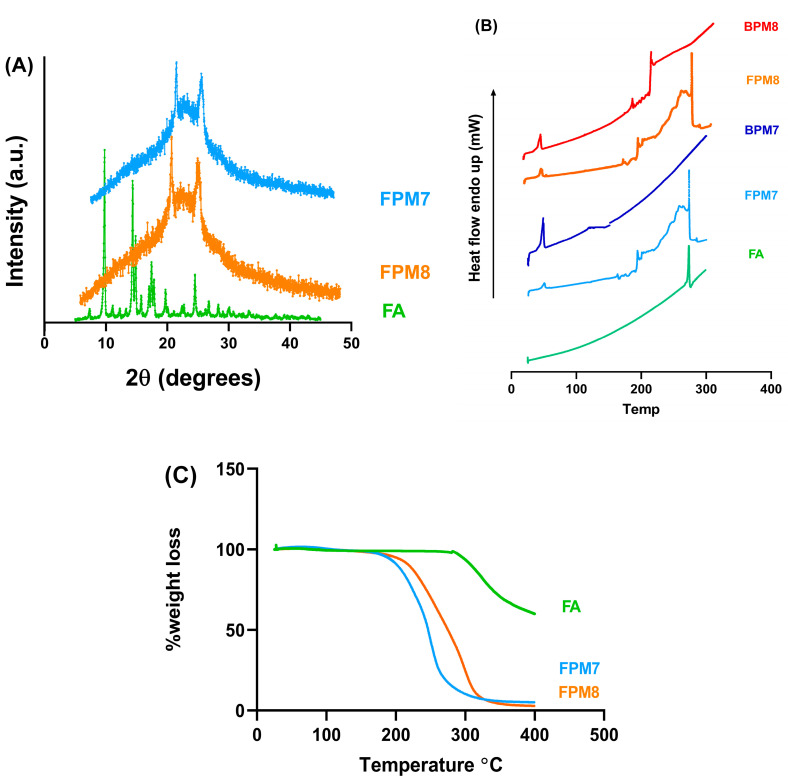
Physical characteristics of fluocinolone acetonide (FA) polymeric micelles: XRD (**A**), DSC (**B**), and TGA (**C**) thermograms.

FPM7 and FPM8 were selected for further investigation with DSC, TGA, and XRD as shown in Figure 5. In comparison to FA, FPM7 and FPM8 were subjected to PXRD analysis to study the crystalline state of FA inside the polymeric micelles. Prominent characteristic peaks of drug crystallization were observed at 20 and 25 (2θ) after incorporating FA into the micellar solution, suggesting the presence of FA in crystalline form (Figure 5A). FA, FPM7, FPM8, and their BPMs were also subjected to DSC studies. A large endothermic peak at 273.37 °C was observed for FA (Figure 5B), which is consistent with the literature and represents FA crystallization. Peaks at 273.32 °C and 274.02 °C were identified for FPM7 and FPM8, respectively, suggesting the existence of crystallized FA inside the polymeric micelles, and supporting the observations from the XRD investigations. Along with the XRD and DSC results, the percentage of weight loss revealed by TGA analysis (Figure 5C) showed no difference between FPM7 and FPM8.

**Figure 6:**
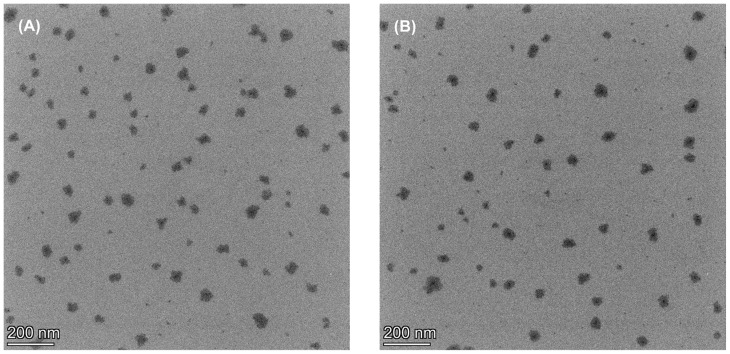
Microscopic morphology photograph of (**A**) FPM7 and (**B**) FPM8 by transmission electron microscope (TEM).

FE-TEM pictures showed irregular nanometer-sized particles in the vesicles. Images of FPM7 and FPM8 showed a similar morphology as shown in Figure 6. The TEM-based size estimates were consistent with the results of DLS particle size analyses (Table 1), which showed that these polymeric micelles surrounded the FA molecule. The results obtained from DSC, TGA, XRD, and FTIR are consistent with the results of morphology and SAXS analysis and allow pharmaceutical researchers to understand the self-rearrangement of FAs and the structures of polymeric micellar carrier systems [36]. 

### 3.6. Ex Vivo Drug Accumulation

**Figure 7:**
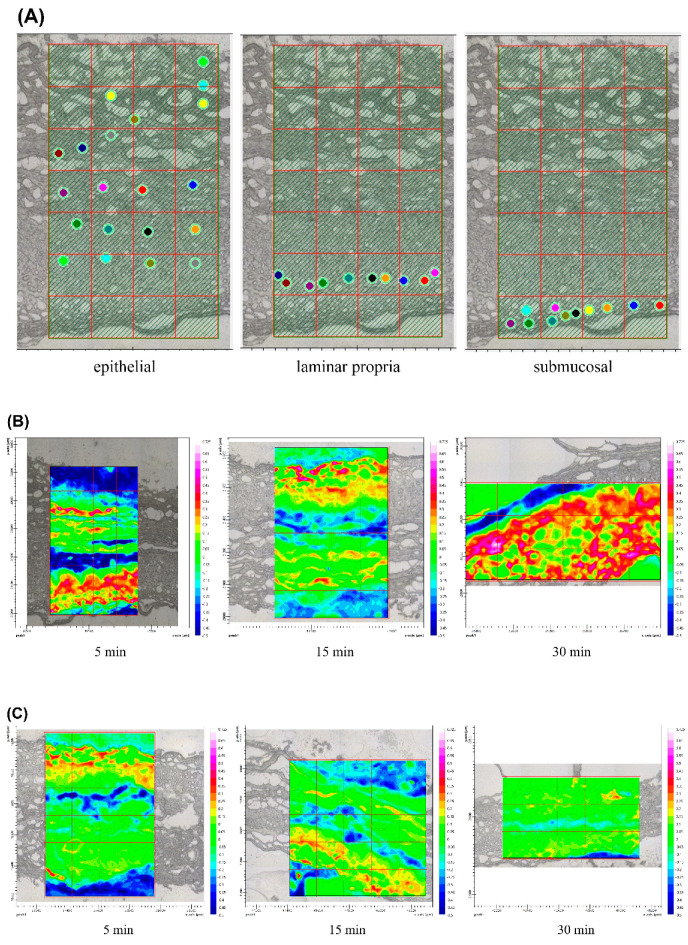
Ex vivo permeation results obtained from the FPA IR images of the epithelial, laminar propria, and submucosa layer of the microtome cross-sectioned barrier membrane, as follows: (**A**) 2D image at initial (blank porcine esophagus), (**B**) 2D image by ATR-FTIR at 5 min, 15 min, and 30 min of cross section of porcine esophagus exposed to FPM7 (**C**) compared to FPM8.

Ex vivo permeation results were obtained from FPA FTIR imaging of the epithelial, laminar propria, and submucosal layers of the microtome cross-sectioned barrier membrane and were differentiated by mapping using oral mucosal histology [37] (see Figure 7). The 3D images of the blank porcine esophagus were used as an initial mucosal sample, prior to treatment (Figure 7A). The 3D images of the cross-section of porcine esophagus exposed to FPM7 were taken with FPA FTIR imaging at 5, 15, and 30 min, and were compared with exposure to FPM8 (Figure 7B,C). At 5 min, the intensity of the epithelium exposed to both FPM7 and FPM8 increased, while all layers of the porcine esophagus showed increased absorbance at 15 min of exposure. At 30 min, FPM7 still showed increased intensity, while tissue exposed to FPM8 showed low intensities throughout the cross-sectional tissue sample. Each layer of the porcine esophagus represented not only the membrane barrier, but also the drug target of action. Corticosteroid, such as FA, reduced inflammation by interacting with receptors on basal cells in the space between the epithelium and lamina propria [3,38]. FPM7 was found to increase in intensity from initial application to 30 min following exposure, suggesting deposition of the formula occurred. In contrast, FPM8 allowed the FA to pass through the mucosal epithelium but did not struct in the layer. 

**Figure 8:**
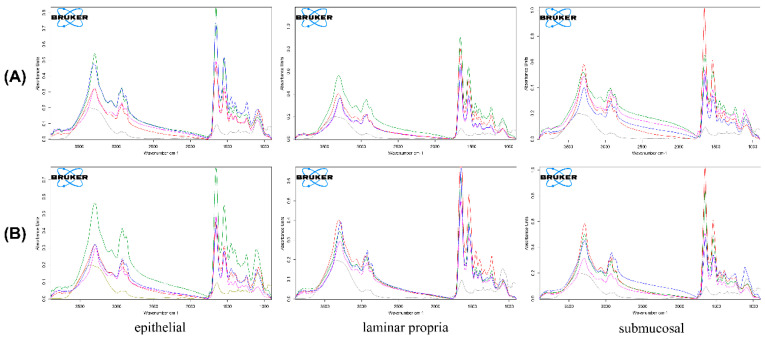
FPA IR of the epithelial, lamina propria, and submucosa layer of the microtome cross-sectioned barrier membrane as fluocinolone acetonide powder (grey line), non-exposure esophagus (red line), and the esophagus exposed to FPM7 (**A**) and FPM8 (**B**) for 5 min (blue line), 15 min (pink line) and 30 min (green line).

FPA FTIR imaging of tissue samples exposed to FPM7 (Figure 8A) and FPM8 (Figure 8B) showed the epithelial, lamina propria, and submucosal layers of the microtome cross-sectioned barrier membrane as blank esophagus (red line) and FA powder (grey line). They were exposed to FPMs for 5 min (blue line), 15 min (pink line), and 30 min (green line). Cross-sections of the epithelium showed that the greatest peak occurred at 30 min for both FPM7 and FPM8. However, at 5 min of permeation, FPM7 was also found in the epithelial layer. Cross-section of the lamina propria revealed a difference in FPM7 and FPM8 permeation. FPM7 showed the highest absorbance peak at 30 min following exposure, while FPM8 had the highest absorbance peak at 5 min. In contrast, the blank mucosal IR spectrum showed that the submucosal layer had the greatest absorption, suggesting that the submucosa did not retain the drug in the membrane, but instead let it pass through to other layers. However, FPM7 still showed peaks after 5, 15, and 30 min of permeation, and FPM8 still showed a peak after 30 min.

Porcine esophageal epithelium is commonly used to represent buccal mucosa in in vitro permeation studies due to similarities in histology and permeability with human oral buccal mucosa [14,39]. FA is a hydrophobic drug with low solubility and high permeability, which is attracted to lipids and repelled by water. Amphiphilic polymers, such as P407 and SPA, were invented to carry hydrophobic drugs like FA and to deliver them to their target sites at the buccal epithelium. The presence of SPA in FPM7 created composite polymeric micelles, increased mucoadhesion [7] and FA lipophilia, and allowed FPM7 to remain in the epithelial layers for longer than FPM8, which contained P407 alone. 

In this study, we intended to design a drug carrier for mouth rinse solution that met several criteria: (1) increased FA solubility; (2) produced an initial burst release; (3) improved drug–mucosal contact time through mucoadhesion; and (4) allowed for drug penetration and retention at the target epithelial site. The composition and ratios of P407:SPA:PEG allowed homogeneous FA to be trapped in polymeric micelles of suitable nanoparticle size, with optimum zeta potential, appropriate viscosity, and good mucoadhesiveness. Moreover, the arrangement of SPA in FPM7 demonstrated high mucoadhesiveness and permeation, and low release in saliva within the first hours of administration, and this is consistent with the hexagonal micellar structure formed from the polymeric surfactant. Refining FA solubility, ensuring initial high permeation, and increasing FA–mucosal contact time allows FAs to penetrate the epithelium and enables the successful development of a polymeric micellar mouth rinse solution for treatment of oral lesions.

Most oral lesions occur at the level of the epithelium, and corticosteroids (particularly FA) are used for wound treatment [3,40]. Thus, improving FA solubility, deposition, and delivery to the basal lamina epithelium using the lipophilic nature of polymeric micelles provides a suitable mouth rinse formulation for FA [6]. The presence of hexagonal polymeric micelles, as well as other physicochemical properties, qualifies FPM as a suitable mouth rinse solution. However, the effectiveness of the formulation and its properties may not be the same as the FPM itself. Clinical trials and investigations of FPM safety and efficacy, including quality of life studies, are therefore required to achieve the goal of sustainable development.

## 4. Conclusions

This study demonstrates the successful development of FA-loaded polymeric micelles composed of two polymers P407 and SPA. The formula of the composite polymers helps to increase mucoadhesion, which is related to drug–mucosal retention time, and allows FA to penetrate and remain in mucosal layers. The hexagonal polymeric micelles’ organization provided a better understanding of the orientation and the impacts on the penetration of lipophilic drug into the mucosa which increases the potential uses of the mouth rinse solution. FPMs could be a superior delivery system as a polymeric micellar mouth rinse dosage form for use as dental material. However, clinical trials (including studies of quality of life) should be carried out prior to development for application.

## Figures and Tables

**Table 1:** 18 different formulations and characterizations were generated by varying the concentrations of P407 (%*w*/*v*), PEG (%*w*/*v*), and SPA (%*w*/*v*) in polymeric micelles containing fluocinolone acetonide (0.1%*w*/*v*) (FPMs).

Rx	P407 (*w*/*v*%)	PEG (*w*/*v*%)	SPA (*w*/*v*%)	Appearance (after Preparation)	Particle Size (nm)	PDI	Zeta potential (mV)	Appearance (at 7 Days)
FPM1	10	15	0.01	Suspension	3827.47 ± 300.36	0.52 ± 0.03	−6.41 ± 1.27	suspension
FPM2	10	15	0	Suspension	2080.8 ± 945.62	0.44 ± 0.04	4.83 ± 9.18	suspension
FPM3	10	10	0.01	Suspension	2341.93 ± 635.85	0.54 ± 0.02	−13.28 ± 1.59	precipitated
FPM4	10	10	0	Suspension	1212.70 ± 886.53	0.42 ± 0.04	6.50 ± 1.15	precipitated
FPM5	10	5	0.01	Suspension	316.63 ± 158.44	0.20 ± 0.01	ND	precipitated
FPM6	10	5	0	Suspension	188.80 ± 92.59	0.21 ± 0.08	ND	precipitated
FPM7	7.5	15	0.01	clear solution	96.20 ± 11.17	0.07 ± 0.004	−21.23 ± 1.70	clear solution
FPM8	7.5	15	0	clear solution	93.50 ± 12.98	0.08 ± 0.002	19.70 ± 1.80	clear solution
FPM9	7.5	10	0.01	clear solution	103.22 ± 36.87	>0.6	ND	precipitated
FPM10	7.5	10	0	clear solution	98.62 ± 46.72	>0.6	ND	precipitated
FPM11	7.5	5	0.01	clear solution	54.03 ± 32.11	>0.6	ND	precipitated
FPM12	7.5	5	0	clear solution	48.14 ± 24.87	>0.6	ND	precipitated
FPM13	5	15	0.01	clear solution	24.12 ± 0.48	0.22 ± 0.08	−3.23 ± 0.47	clear solution
FPM14	5	15	0	clear solution	12.34 ± 0.12	0.24 ± 0.11	0.11 ± 0.70	clear solution
FPM15	5	10	0.01	Precipitated	ND	ND	ND	precipitated
FPM16	5	10	0	Precipitated	ND	ND	ND	precipitated
FPM17	5	5	0.01	Precipitated	ND	ND	ND	precipitated
FPM18	5	5	0	Precipitated	ND	ND	ND	precipitated

ND represented not determined.

**Table 2:** The effects of independent variables, such as poloxamer 407 (P407), PEG400 (PEG), and sodium polyacrylate (SPA) on micelles containing 0.1% fluocinolone acetonide (FA), in relation to viscosity (cps), mucoadhesiveness (%), drug release (%), and drug permeation (%). Mucoadhesiveness was determined by the percentage of FA interacting with mucin at 0 and 15 min. Drug release and drug permeation were measured at initial 5 min.

Rx	Viscosity (cps)	%Mucoadhesive at 0 min	%Mucoadhesive at 15 min	%Drug Release	%Drug Permeation
FPM1	41.8 ± 0.35	6.2 ± 0.88	15.8 ± 2.63	2.47 ± 0.001	14.58 ± 0.03
FPM2	41.2 ± 0.35	4.9 ± 2.27	15.5 ± 1.62	1.64 ± 0.004	9.15 ± 0.001
FPM7	20.4 ± 0.02	38.2 ± 5.88	82.4 ± 0.04	1.70 ± 0.001	17.76 ± 0.001
FPM8	19.8 ± 0.60	41.4 ± 7.16	89.0 ± 5.93	0.76 ± 0.003	12.37 ± 0.03
FPM13	10.6 ± 0.69	17.8 ± 4.90	57.1 ± 11.84	0.07 ± 0.000	17.54 ± 0.03
FPM14	10.8 ± 0.60	8.6 ± 1.69	51.4 ± 11.49	0.01 ± 0.000	15.74 ± 0.04

ND represented not determined.

**Table 3:** Drug release over 8 h according to zero-order, first-order, Higuchi, and Korsmeyer-Peppas release kinetic models.

Kinetic Models	Zero-Order		First-Order		Higuchi		Korsmeyer-Peppas		
	K_0_	r^2^	K_1_	r^2^	K_H_	r^2^	K_KP_	n	r^2^
Formulation									
FPM1	0.0610	0.9933	0.0025	0.9464	1.4362	0.9191	0.058	1.015	0.9902
FPM2	0.0633	0.9949	0.0028	0.9364	1.4923	0.9243	0.058	1.014	0.9902
FPM7	0.1095	0.9710	0.0035	0.9130	2.5346	0.8694	0.074	1.003	0.9922
FPM8	0.0732	0.9944	0.0027	0.8979	1.7334	0.9323	0.007	1.459	0.9958
FPM13	0.0721	0.9979	0.0038	0.8261	1.7084	0.9368	0.024	1.173	0.9993
FPM14	0.0922	0.9963	0.0049	0.7507	2.1798	0.9300	0.024	1.216	0.9991

**Table 4:** Drug permeation over 60 min according to zero-order, first-order, Higuchi, and Korsmeyer-Peppas models.

Kinetic Models	Zero-Order		First-Order		Higuchi		Korsmeyer-Peppas		
	K_0_	r^2^	K_1_	r^2^	K_H_	r^2^	K_KP_	n	r^2^
Formulation									
FPM1	1.1750	0.9688	0.0125	0.8683	12.129	0.9913	5.191	0.671	0.9918
FPM2	1.1113	0.9604	0.0142	0.8073	11.266	0.9480	2.989	0.780	0.9650
FPM7	1.0338	0.9132	0.0115	0.7463	10.942	0.9824	6.293	0.578	0.9896
FPM8	0.9142	0.9389	0.0115	0.7751	9.5896	0.9921	7.694	0.562	0.9813
FPM13	0.7322	0.8755	0.0094	0.7894	7.7891	0.9515	8.189	0.485	0.9691
FPM14	0.8113	0.9024	0.0092	0.7828	8.6120	0.9764	9.570	0.475	0.9864

**Table 5:** The pH of and percentage remaining of fluocinolone acetonide (FA) following storage at 4°C, 25°C, or 45 °C (expressed as mean ± standard deviation, n = 6).

Rx	Day	pH	Percentage of Drug Content (%)		
			4 ± 2 °C	25 ± 2 °C	45 ± 2 °C
1	0	7.42 ± 0.01	100.5 ± 0.01	100.5 ± 0.01	100.5 ± 0.01
	7		101.2 ± 0.01	100.2 ± 0.01	101.2 ± 0.01
	30		103.0 ± 0.04	97.6 ± 0.06 ^a^	91.8 ± 0.01 ^a^
2	0	7.42 ± 0.01	100.9 ± 0.01	100.9 ± 0.02	100.9 ± 0.02
	7		103.2 ± 0.09	101.1 ± 0.04	99.12 ± 0.01
	30		97.8 ± 0.07 ^b^	91.7 ± 0.03 ^b^	91.0 ± 0.04 ^b^
7	0	7.42 ± 0.01	104.1 ± 0.01	101.4 ± 0.01	104.1 ± 0.01
	7		100.6 ± 0.04	100.3 ± 0.02	102.3 ± 0.02
	30		100.4 ± 0.03	100.0 ± 0.01	100.4 ± 0.01
8	0	7.42 ± 0.01	100.3 ± 0.03	100.3 ± 0.03	100.3 ± 0.03
	7		100.2 ± 0.01	100.2 ± 0.01	100.6 ± 0.03
	30		100.0 ± 0.04	100.1 ± 0.01	100.0 ± 0.01
13	0	7.42 ± 0.01	100.4 ± 0.01	100.4 ± 0.01	100.4 ± 0.01
	7		100.2 ± 0.02	99.4 ± 0.05	95.2 ± 0.07 ^c^
	30		100.1 ± 0.01	98.5 ± 0.08 ^c^	87.4 ± 0.09 ^c^
14	0	7.42 ± 0.01	100.3 ± 0.01	100.3 ± 0.01	100.3 ± 0.01
	7		102.2 ± 0.04	100.2 ± 0.01	87.9 ± 0.07 ^d^
	30		95.3 ± 0.06 ^d^	97.4 ± 0.08 ^d^	59.0 ± 0.08 ^d^

^a^ Represented *p* > 0.005 compared to formulation FPM1 at day 0; ^b^ represented *p* > 0.005 compared to formulation FPM2 at day 0; ^c^ represented *p* > 0.005 compared to formulation FPM13 at day 0; and ^d^ represented *p* > 0.005 compared to formulation FPM14 at day 0.

## Data Availability

Not applicable.
